# Editorial: Biomarkers in autoimmune diseases of the central nervous system

**DOI:** 10.3389/fimmu.2023.1266953

**Published:** 2023-08-18

**Authors:** Yin-Xi Zhang, Hong-Hao Wang, Shou-Gang Guo, Long-Jun Wu, Mei-Ping Ding

**Affiliations:** ^1^ Department of Neurology, Second Affiliated Hospital, School of Medicine, Zhejiang University, Hangzhou, China; ^2^ Department of Neurology, Guangzhou First People’s Hospital, School of Medicine, Southern China University of Technology, Guangzhou, China; ^3^ Department of Neurology, Shandong Provincial Hospital Affiliated to Shandong First Medical University, Shandong Academy of Medical Sciences, Jinan, China; ^4^ Department of Neurology, Mayo Clinic, Rochester, MN, United States

**Keywords:** biomarker, autoimmune disease, central nervous system, inflammatory demyelinating disease, autoimmune encephalitis

Autoimmune diseases of the central nervous system (CNS) represent a group of complex and disabling disorders characterized by the immune system mistakenly targeting the brain and spinal cord. This results in structure damage and functional impairment. The pathogenesis of these diseases involves immune cells, autoantibodies and immune molecules directly or indirectly attacking the CNS, leading to neuronal or axonal injury, myelin loss and other neuropathological changes. Although CNS autoimmune diseases account for a small portion of neurological disorders, patients may exhibit extensive involvement and various manifestations, posing significant challenges in diagnosis and treatment. In the past two decades, there has been a rapid expansion in the understanding of CNS autoimmune diseases, particularly CNS inflammatory demyelinating diseases and autoimmune encephalitis. While notable discoveries have shed light on the autoimmune basis of these conditions, the exact pathogenesis remains unclear, and further in-depth research is needed.

Biomarkers are of great value in our understanding and management of CNS autoimmune diseases. They reflect the presence, nature, and intensity of certain immune responses triggered by both genetic and environmental factors. Biomarkers are of great importance in clinical diagnosis, estimating disease risk or prognosis, evaluating disease severity, and monitoring treatment response and disease progression (1). For instance, the detection of disease-specific antibodies aids in accurate diagnosis and precise treatment. Furthermore, the identification of diverse biomarkers holds the potential to advance personalized medicine.

To provide a platform for sharing the latest research advances in this field, we have organized this Research Topic. The Research Topic comprises 21 manuscripts, including 13 original research articles, 4 brief research reports, 1 review, 1 meta-analysis and systematic review and 2 case reports. These papers have broadened the contemporary knowledge and understanding of biomarkers of autoimmune CNS disorders. In this Editorial, we highlight the representative articles contributing to this Research topic and summarize their main findings.

CNS inflammatory demyelinating diseases are a category of autoimmune-mediated disorders sharing the basic pathological hallmark of myelin loss and neuroinflammation. These diseases occur throughout the world and preferentially affect young adults, with multiple sclerosis (MS), neuromyelitis optica spectrum disorder (NMOSD), and myelin oligodendrocyte glycoprotein (MOG) antibody-associated disease (MOGAD) being the main representatives (2, 3). CNS inflammatory demyelinating diseases have distinct clinical characteristics, and involve biomarkers with important clinical implication. Liu et al. analyzed circulating antigen-specific memory T cell subsets, to explore their association with disease activity of MS. Their findings revealed positive regulatory roles for CD8+ memory T cell populations in MS, which established a valuable foundation for identifying potential serological biomarkers and exploring novel treatment approaches. A study by Karimi et al. focused on the regulatory transcriptional gene network underlying MS. The results demonstrated that the LASP1, S100A6 and TUBA1C genes were most likely to play a biological role in the development of MS and might serve as potential diagnostic and therapeutic biomarkers. Yadav et al. conducted the study using a humanized spontaneous experimental autoimmune encephalomyelitis model to investigate the underlying biology of MS-associated gut inflammation. They observed that gut infiltration of Th17 cells and recruitment of neutrophils were linked with the development of gut dysbiosis and intestinal inflammation, and suggested that fecal Lcn-2 level was a sensitive biological indicator for gut dysbiosis in MS. In addition, Zhou et al. recruited 30 Eastern patients with MS and comprehensively evaluated the cerebral blood flow (CBF) features using the arterial spin labeling technique and their relationship with multiple clinical parameters for the first time. The authors concluded that CBF could be a potential quantitative neuroimaging marker associated with disease severity. Miyamoto et al. performed a retrospective analysis of serum complement factors in 21 patients with NMOSD and 25 patients with Guillain-Barré syndrome. The study revealed that complement biomarkers (e.g., Ba, sC5b-9, and complement factor H) in peripheral blood could contribute to the pathogenesis and pathological status of NMOSD. Shi et al. collected 90 blood samples from 59 patients with NMOSD and 31 healthy controls, aiming to assess the correlation between granzyme B (GzmB) levels in CD8+T cells and clinical characteristics. They found the involvement of GzmB-expressing CD8+ T cells in the inflammatory response in NMOSD, which could be considered a possible biomarker for therapeutic effectiveness and disability progression. Chen et al. compared the different immunological mechanisms between aquaporin 4 antibody-positive optic neuritis (ON) and MOG antibody-positive ON based on transcriptomics analysis of patients’ whole blood, providing novel insights into the pathogenesis of these two diseases. Moreover, Sun C. et al. retrospectively examined the neuroimaging and clinicopathological differences between tumefactive demyelinating lesions and sentinel lesions of primary central nervous system lymphoma, designing to determine relevant biomarkers and improve early accurate diagnosis.

Compared with CNS inflammatory demyelinating diseases, autoimmune encephalitis is a relatively new field of research but recently becomes an active research hotspot in CNS neuroimmunology. Autoimmune encephalitis is an umbrella term for the non-infectious, immune-mediated inflammation of the brain parenchyma, in which neural antibodies can be found in a large proportion of patients (4). Neuronal surface antibody-associated autoimmune encephalitis is the most common subgroup, mainly including anti-N-methyl-D-aspartate receptor (NMDAR) encephalitis and anti-leucine-rich glioma-inactivated 1 (LGI1) encephalitis (5). Shao et al. explored the coagulation function in patients with anti-NMDAR encephalitis, identifying serum D-dimer and neutrophil levels as effective predictors of disease severity for anti-NMDAR encephalitis. Ma et al. conducted a meta-analysis and systematic review, analyzing the concentrations of cytokines/chemokines in the unstimulated cerebrospinal fluid (CSF) or serum of patients with anti-NMDAR encephalitis. Their findings highlighted the involvement of multiple immune cell interactions mediated by cytokines/chemokines in the central immune response, with T cells playing a pivotal role in the immunopathogenesis of anti-NMDAR encephalitis. Li J. et al. examined chitinase-3 likeprotein-1 (CHI3L1) and its correlation with modified Rankin Scale score. They concluded that CHI3L1 level in CSF was associated with the severity and outcome of anti-LGI1 encephalitis. Another study by Zhao-Fleming et al. characterized the cardiac arrythmias among patients with anti-LGI1 encephalitis, emphasizing the importance of identifying this phenomenon despite the rarity and generally favorable prognosis. Hara et al. investigated lymphocyte subset analyses of B cells and circulating T follicular helper cells (cTfh) in patients with autoimmune encephalitis with seizures. The results revealed that elevated frequency of plasmablasts and inducible T-cell co-stimulator-expressing cTfh17 shift in peripheral blood mononuclear cells might provide a new indicator for the presence of antibodies in patients with autoimmune encephalitis. Additionally, Sun Y. et al. enrolled 10 patients with anti-amphiphysin encephalitis and described the clinical and paraclinical characteristics, treatment, and prognostic predictors. Zhu et al. reported a case of anti-neurexin-3α-associated autoimmune encephalitis secondary to contrast-induced encephalopathy. Li Y. et al. presented a patient with the dual positivity of anti-NMDAR antibody and anti-metabotropic glutamate receptor 5 antibody, along with bilateral ovarian teratomas and reversible splenial lesion syndrome. As the most important diagnostic biomarker for autoimmune encephalitis, precise antibody test and comprehensive result interpretation are crucial. Muñoz-Sánchez et al. assessed the clinical performance of two indirect immunofluorescent cell-based assays (IIF-CBA) using paired serum/CSF in a large cohort of patients with anti-LGI1 encephalitis. They pointed out that both serum and CSF samples should be examined if using a commercial IIF-CBA for antibody confirmation to reduce false negative results in suspected anti-LGI1 encephalitis. Zong et al. evaluated the anti-glutamate decarboxylase 65 (GAD65) antibody levels measuring by different detection methods and reconfirmed that GAD65 antibody levels were significantly higher in patients with neuropsychiatric disease than in patients with diabetes. Furthermore, Nagata et al. addressed the clinical relevance of currently available commercial rat brain immunohistochemistry and the immunostaining patterns of neuronal surface antigens. The authors stated that tissue-based assay is clinically helpful for screening neuronal surface antibody and glial fibrillary acidic protein antibody, and neuronal surface antigen specific immunoreactivity can be regarded as a useful biomarker of autoimmune encephalitis.

Finally, the well-rounded review of this Research Topic by Zhang et al. summarized the biomarkers in autoimmune diseases of the CNS and their potential clinical significance and application prospects. These biomarkers were usually classified into diagnostic, drug monitoring and safety and outcome predictive purpose based on clinical need, containing CNS injury markers, humoral markers, cytokines and cell markers in serum or CSF. They concluded that promising biomarkers had a significant impact on early intervention and prevention of future disability.

In summary, the collection of articles in this Research Topic expands the current knowledge regarding biomarkers in autoimmune diseases of the CNS. These studies provide novel scientific evidence and highlight recent progress in this continually evolving field.

## Author contributions

Y-XZ: Conceptualization, Data curation, Formal Analysis, Investigation, Writing – original draft, Writing – review & editing. H-HW: Conceptualization, Data curation, Formal Analysis, Investigation, Supervision, Validation, Writing – review & editing. S-GG: Conceptualization, Data curation, Formal Analysis, Investigation, Supervision, Validation, Writing – review & editing. L-JW: Conceptualization, Data curation, Formal Analysis, Investigation, Supervision, Validation, Writing – review & editing. M-PD: Conceptualization, Data curation, Formal Analysis, Investigation, Supervision, Validation, Writing – review & editing.
